# Applying GRADE-CERQual to qualitative evidence synthesis findings—paper 3: how to assess methodological limitations

**DOI:** 10.1186/s13012-017-0690-9

**Published:** 2018-01-25

**Authors:** Heather Munthe-Kaas, Meghan A. Bohren, Claire Glenton, Simon Lewin, Jane Noyes, Özge Tunçalp, Andrew Booth, Ruth Garside, Christopher J. Colvin, Megan Wainwright, Arash Rashidian, Signe Flottorp, Benedicte Carlsen

**Affiliations:** 1 Norwegian Institute of Public Health, Oslo, Norway; 2 UNDP/UNFPA/ UNICEF/WHO/World Bank Special Programme of Research, Development and Research Training in Human Reproduction, Department of Reproductive Health and Research, World Health Organization, Geneva, Switzerland; 3 Health Systems Research Unit, South African Medical Research Council, Cape Town, South Africa; 4 School of Social Sciences, Bangor University, Bangor, UK; 5 School of Health and Related Research (ScHARR), University of Sheffield, Sheffield, UK; 6 European Centre for Environment and Human Health, University of Exeter Medical School, Exeter, UK; 7 Division of Social and Behavioural Sciences, School of Public Health and Family Medicine, University of Cape Town, Cape Town, South Africa; 8 Department of Health Management and Economics, School of Public Health, Tehran University of Medical Sciences, Tehran, Iran; 9 Information, Evidence and Research Department, Eastern Mediterranean Regional Office, World Health Organization, Cairo, Egypt; 10 Uni Research Rokkan Centre, Bergen, Norway

**Keywords:** Qualitative research, Qualitative evidence synthesis, Systematic review methodology, Research design, Methodology, Confidence, Guidance, Evidence-based practice, Methodological limitations, GRADE, Critical appraisal

## Abstract

**Background:**

The GRADE-CERQual (Confidence in Evidence from Reviews of Qualitative research) approach has been developed by the GRADE (Grading of Recommendations Assessment, Development and Evaluation) Working Group. The approach has been developed to support the use of findings from qualitative evidence syntheses in decision-making, including guideline development and policy formulation.

CERQual includes four components for assessing how much confidence to place in findings from reviews of qualitative research (also referred to as qualitative evidence syntheses): (1) methodological limitations, (2) coherence, (3) adequacy of data and (4) relevance. This paper is part of a series providing guidance on how to apply CERQual and focuses on CERQual’s methodological limitations component.

**Methods:**

We developed the methodological limitations component by searching the literature for definitions, gathering feedback from relevant research communities and developing consensus through project group meetings. We tested the CERQual methodological limitations component within several qualitative evidence syntheses before agreeing on the current definition and principles for application.

**Results:**

When applying CERQual, we define methodological limitations as the extent to which there are concerns about the design or conduct of the primary studies that contributed evidence to an individual review finding. In this paper, we describe the methodological limitations component and its rationale and offer guidance on how to assess methodological limitations of a review finding as part of the CERQual approach. This guidance outlines the information required to assess methodological limitations component, the steps that need to be taken to assess methodological limitations of data contributing to a review finding and examples of methodological limitation assessments.

**Conclusions:**

This paper provides guidance for review authors and others on undertaking an assessment of methodological limitations in the context of the CERQual approach. More work is needed to determine which criteria critical appraisal tools should include when assessing methodological limitations. We currently recommend that whichever tool is used, review authors provide a transparent description of their assessments of methodological limitations in a review finding. We expect the CERQual approach and its individual components to develop further as our experiences with the practical implementation of the approach increase.

**Electronic supplementary material:**

The online version of this article (10.1186/s13012-017-0690-9) contains supplementary material, which is available to authorized users.

## Background

The GRADE-CERQual (Confidence in Evidence from Reviews of Qualitative research) approach has been developed by the GRADE (Grading of Recommendations Assessment, Development and Evaluation) Working Group. The approach has been developed to support the use of findings from qualitative evidence syntheses in decision-making, including guideline development and policy formulation. GRADE-CERQual (hereafter referred to as CERQual) includes four components for assessing how much confidence to place in findings from reviews of qualitative research (also referred to as qualitative evidence syntheses): (1) methodological limitations, (2) coherence, (3) adequacy of data and (4) relevance. This paper focuses on one of these four components: methodological limitations.

When carrying out a CERQual assessment, we define methodological limitations as the extent to which there are concerns about the design or conduct of the primary studies that contributed evidence to an individual review finding. Where the primary studies underlying a review finding are assessed as having methodological limitations, and these are considered to have a clear/direct impact on the review finding, we may be less confident that the review finding reflects the phenomenon of interest [1].

When assessing methodological limitations, our goal is not to judge whether some absolute standard of methodological quality has been achieved, but rather to indicate concerns where any methodological limitations have been identified as serious enough to lower our confidence in the review finding. The methodological limitations component is analogous to the risk of bias domain used in the GRADE approach for findings from systematic reviews of effectiveness [2, 3].

## Aim

**Fig. 1:**
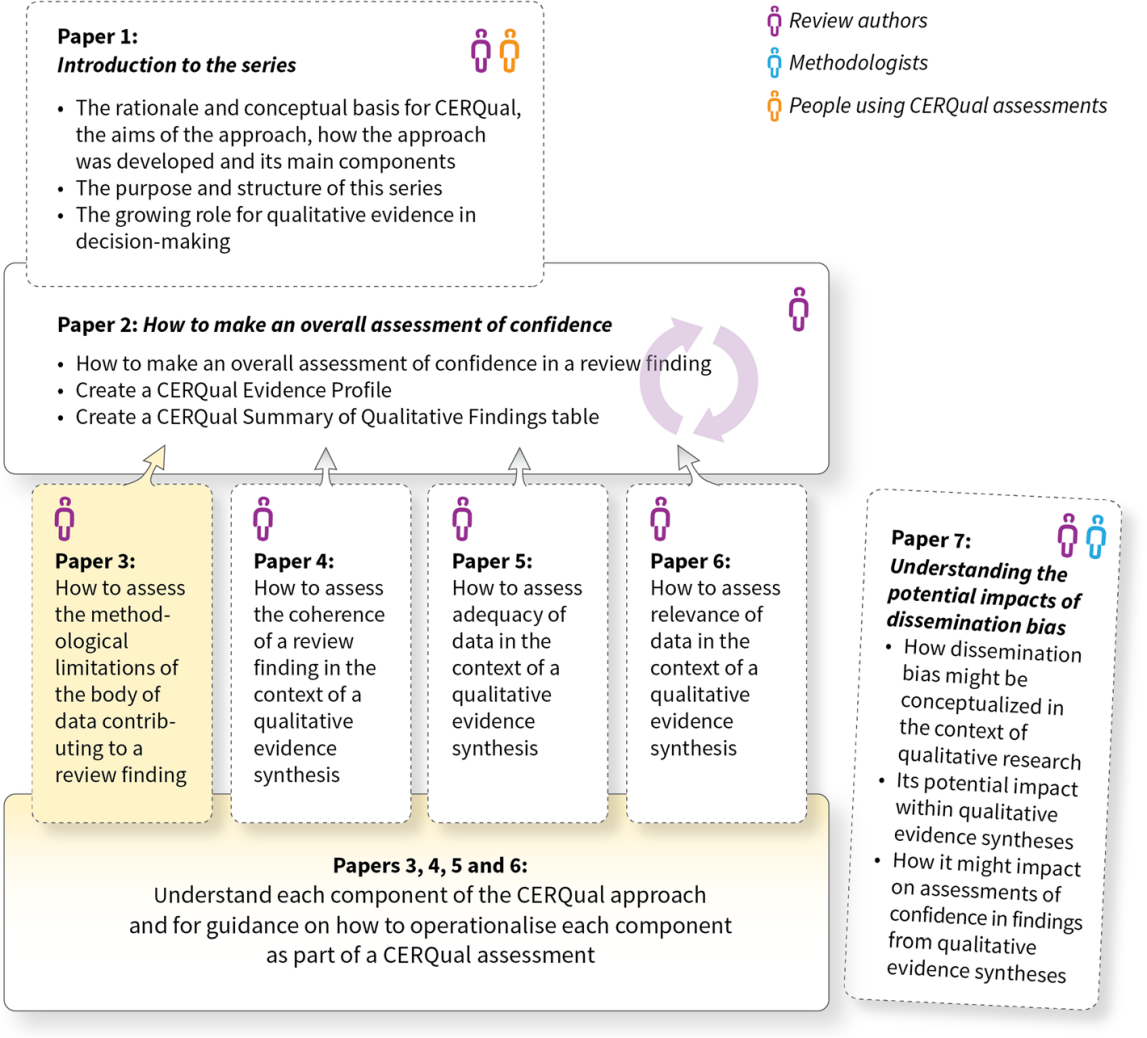
Overview of the GRADE-CERQual series of papers

The aim of this paper, part of a series (Fig. 1), is to describe what we mean by methodological limitations of the body of data (data from included primary studies) contributing to a review finding in the context of a qualitative evidence synthesis and to give guidance on how to operationalise this component in the context of a review finding as part of the CERQual approach. This paper should be read in conjunction with the papers describing the other three CERQual components [4–6] and the paper describing how to make an overall CERQual assessment of confidence and create a summary of qualitative findings table [7]. Key definitions for the series are provided in Additional file 1.

## How CERQual was developed

The initial stages of the process for developing CERQual, which started in 2010, are outlined elsewhere [1]. Since then, we have further refined the current definitions of each component and the principles for application of the overall approach using a number of methods. When developing CERQual’s methodological limitations component, we undertook informal searches of the literature, including Google and Google Scholar for relevant critical appraisal tools, and for definitions and discussion papers related to the concept of methodological quality in the context of qualitative research. We carried out similar searches for the other three components. We presented an early version of the CERQual approach in 2015 to a group of methodologists, researchers and end users with experience in qualitative research, GRADE or guideline development. We further refined the approach through training workshops, seminars and presentations during which we actively sought, collated and shared feedback, by facilitating discussions of individual CERQual components within relevant organisations, through applying the approach within diverse qualitative evidence syntheses [8–18] and through supporting other teams in using CERQual [19, 20]. As far as possible, we used a consensus approach in these processes. We also gathered feedback from CERQual users through an online feedback form and through short individual discussions with members of the review teams. The methods used to develop CERQual are described in more detail in the first paper in this series [21].

## Assessing methodological limitations

### Methodological limitations in the context of findings from qualitative evidence syntheses

The methodological approaches used in a primary study may have consequences for how much we can trust the findings from that study. Where there are concerns regarding the appropriateness of these approaches (e.g. data collection or analysis methods), or how the studies were conducted, study findings may be produced that are not an adequate representation of the phenomenon of interest. For example, we may have less trust in findings from a study where participants were recruited in a manner that did not fully address the aims of the research or where the data analysis methods were not appropriate for the study design.

One or more studies contribute data to each review finding in a qualitative evidence synthesis, and these data make up the body of data for a review finding. The methodological limitations of the body of data supporting a review finding are assessed as a whole to identify whether or not any methodological weaknesses within individual studies impact our confidence in a review finding. The methodological limitations for each review finding must be assessed separately since different studies contribute varying amounts of data to each review finding, and methodological quality issues may have varying impacts on different review findings. For example, the same set of studies may contribute data to many review findings. However, individual features of study design may have implications for some of those review findings, but not necessarily other review findings. Methodological limitations of the body of data may weaken our overall assessment of confidence in the review findings to which these studies contribute. See Table 1 for the examples of review findings with concerns regarding methodological limitations.

**Table 1:** CERQual assessments of methodological limitations in the context of a review finding – Examples

Example 1. No or very minor concerns
A qualitative evidence synthesis examined mistreatment of women during childbirth in medical facilities [11]*. One review finding dealt with women’s preferences: “Women preferred female to male practitioners.” Nine studies contributed to this review finding. All of these studies were assessed as having methodological limitations concerning reflexivity (the individuals collecting and analysing the data were also providing healthcare during childbirth). This body of evidence supporting the review finding was assessed as having no or minor concerns regarding methodological limitations because the dual role of researcher and healthcare provider was not seen to affect this stated preference.
Example 2. Minor concerns
A qualitative evidence synthesis explored parents’ and informal caregivers’ views and experiences regarding communication about childhood vaccinations [8]*. One finding was that “parents liked to receive information about vaccination before the baby was born for reasons such as fatigue and time limitations for reading about vaccination after delivery.” Five studies contributed data to this finding. None of the studies used methods such as triangulation or respondent validation to check the credibility of their findings. The authors concluded that there were “minor concerns regarding methodological limitations due to a lack of discussion by primary authors regarding credibility of the data.”
Example 3. Moderate concerns
Another review finding from the qualitative synthesis examining mistreatment of women during childbirth [11] was considered to be of a sensitive nature since it discussed the women’s bodies and directly criticized specific types of caregivers: “Some women complained of lack of understanding and rough treatment from caregivers, specifically during vaginal and abdominal exams.” Twenty studies contributed data to this review finding. Five studies were assessed as having methodological limitations related to how the data was collected (it is not clear that the authors obtained informed consent) and related to researcher reflexivity (the individuals collecting the data were also providing healthcare during childbirth). An additional fifteen studies were assessed as having methodological limitations only related to the reflexivity of the researcher (the researchers’ role was either unclear, or they were also healthcare providers in maternity wards). The body of evidence contributing to the review finding was assessed as having moderate concerns regarding methodological limitations due to concerns regarding reflexivity –the researchers’ dual role as health providers and caregivers during childbirth was seen as potentiallyhaving an effect on what the women would report afterwards regarding their experiences.
Example 4. Serious concerns
Another finding from the synthesis on communication about childhood vaccinations was that “some parents vaccinated their children because of perceived pressure from the health services” [8]. Seven studies contributed data to this finding. Three of these studies did not describe data collection methods in detail, lacked discussion of researcher reflexivity, and described inappropriate analysis methods (counting). Four studies did not present sufficient data to support the findings, and did not report on how the data was collected or analysed. The authors concluded that there were “serious concerns regarding methodological limitations due to data collection and analysis methods and a lack of researcher reflexivity.”

*These findings have been adapted from the original qualitative evidence synthesis to highlight issues regarding methodological limitations

### Critical appraisal of qualitative research

The extent to which it is possible or appropriate to critically appraise the methodological quality of qualitative research is contested among researchers in the field [22–25]. However, the starting point for the CERQual approach is that there is a need for ‘clear evaluative criteria that are responsive to the unique nature of qualitative inquiry’ ([26] p. 113).

Despite the existence of more than 50 guidelines for assessing the quality of qualitative research [27], there is no agreement on the best approach for assessing the methodological quality of primary qualitative studies. Even where essential criteria for assessing methodological quality have been agreed upon, there are challenges related to the definitions underlying these criteria, and how much importance should be given to them within a critical appraisal tool [27] (p. 151).

The methodological limitations component of the CERQual approach requires some systematic and transparent approach for identifying methodological weaknesses of individual studies. Critical appraisal tools can help us to identify such weaknesses. We are, however, dependent on the quality and completeness of the individual study reports. The Cochrane Qualitative and Implementation Methods Group recommends that review authors consider a number of issues when choosing a tool to assess methodological strengths and limitations of qualitative studies, including the type of designs or methods selected to address the review question and the included primary studies [28]. In addition, the group advises using tools that privilege assessment of methodological strengths and limitations over the quality of reporting [29]. In the context of CERQual, we take a similar approach, and currently recommend that review authors use an approach that fits their review question and synthesis methods, with which they are familiar and comfortable using, and that focuses on methodological strengths and limitations. Regardless of the approach chosen, the review authors should provide a detailed and transparent assessment for each element of the tool. In line with Cochrane Qualitative and Implementation Methods Group guidance, we do not recommend using reporting guidelines as proxies for quality appraisal tools (see Table 2). To date, review authors using the CERQual approach have primarily used the Critical Appraisal Skills Programme (CASP) checklist or an adapted version of it [30]. Research is underway to examine which elements of critical appraisal are key for assessing the quality of research in the context of qualitative evidence synthesis and for use in the CERQual approach. See Table 2 for an outline of areas where further work is needed with respect to critical appraisal tools for qualitative research.

**Table 2:** Areas where further work is needed – Critical appraisal tools

Despite the existence of a variety of checklists and tools there is no agreement on the best approach to assessing the methodological limitations of qualitative studies [27, 31]. Furthermore, in general, the criteria included in existing critical appraisal tools for qualitative studies are considered inadequate when applying CERQual as they are not based on evidence or explicit hypotheses regarding the relationships between components of qualitative study design and conduct and the trustworthiness of the study findings. We plan to undertake further work to locate any existing evidence that can help us identify the most important elements of a critical appraisal tool when used in the context of CERQual. We may then develop a critical appraisal tool for use with CERQual.	

## Guidance on how to assess methodological limitations in the context of a review finding 

**Fig. 2:**
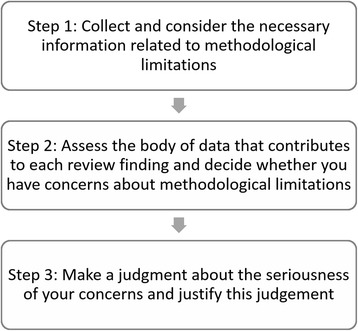
Steps when assessing methodological limitations

The steps taken when assessing methodological limitations are shown in Fig. 2 and detailed below.

### Step 1: collect and consider the necessary information related to methodological limitations

To assess methodological limitations of the body of data contributing to a review finding, you first need to choose an appropriate critical appraisal tool to assess the methodological strengths and limitations of the primary studies contributing data to the review finding. Regardless of the chosen tool, you will need to collect detailed information regarding the methods of data collection and analysis used in each study, as well as other aspects covered by the critical appraisal tool that you have chosen. The level of detail reported on the conduct of the included studies may vary greatly depending on the study design, the topic/field, type of publication or journal specifications.

When applying CERQual to a review that you have conducted, you will normally have gathered this information during data extraction, as this is a standard part of the review process. When doing so, you should present and explain in detail the assessments of each criterion within the critical appraisal tool for each primary study. Some review teams choose to present this as a matrix of methodological limitations of included studies. However, if you are applying CERQual to findings from somebody else’s review, you will need to have access to their critical appraisal assessments of the included studies, which are often published as part of the review. Where these assessments are not available, you may need to go directly to the included primary studies and assess the methodological strengths and limitations for each included study yourself. For more information on applying CERQual to findings from someone else’s review, see [7].

#### Issues to consider in this step

Consider the specific study design and the research question when choosing and applying a critical appraisal tool to assess the methodological strengths and limitations of the included studies.

Remember that as part of the CERQual assessment, the methodological limitations for each review finding will be examined separately. Therefore, you will need to provide a detailed explanation for your assessment of each component of the critical appraisal tool, rather than ticking ‘yes’ or ‘no’ on a checklist.

### Step 2: assess the body of data that contributes to each review finding and decide whether you have concerns about methodological limitations

Once you have carried out your critical appraisal of the included studies, you can start to assess whether you have concerns regarding any methodological limitations of the body of data supporting each review finding.

#### Issues to consider in this step

Some methodological strengths and weaknesses may be important for some review findings but not others. For instance, many critical appraisal tools ask you to assess whether the method of data collection was appropriate, but while methods such as focus groups may be inappropriate as a method of collecting data for some sensitive topics, they would not be considered inappropriate for other less sensitive topics. You should reflect on whether the review finding is particularly affected by any methodological limitations identified in contributing studies. You may find it helpful for the review team to meet before starting an assessment of methodological limitations to discuss and agree upon any specific issues likely to affect review findings, such as (but not limited to) privacy/sensitivity of issues, risk to participants, social desirability, the presence of observation that might affect ‘authentic’ behaviour (e.g. Hawthorne effect) and researcher effects.

Consider each study’s relative contribution to the review finding. For instance, if one study with serious methodological limitations contributes most of the data to a review finding, you may consider indicating serious concerns with methodological limitations, regardless of the methodological limitations of the other contributing studies.

Recognise that not all methodological limitations raise the same level of concerns. Consider the types of methodological limitations identified, and to what degree your concerns regarding those limitations may affect your overall confidence in the review finding.

Consider if the assessment of methodological limitations is impacted by absent information regarding how a study was conducted and if the lacking information is important to the review finding. Consider contacting the authors of the primary studies if essential reporting information is missing.

Be aware of whether the critical appraisal tool addresses issues related to one of the other three components of CERQual. For example, the critical appraisal tool may prompt you to examine the richness of the data presented (which overlaps with CERQual’s adequacy component) or whether the study findings would be applicable to the review context (which overlaps with CERQual’s relevance component). You should indicate in your assessment of methodological limitations that these issues have been included, and consider this when assessing the other components. Alternatively, you may choose to leave out questions in a critical appraisal tool that are covered by other CERQual components.

### Step 3: make a judgement about the seriousness of your concerns and justify this judgement

Once you have assessed methodological limitations for each review finding, you should categorise any concerns that you have identified as either of the following:

- No or very minor concerns
- Minor concerns
- Moderate concerns
- Serious concerns

You should begin with the assumption that there are no concerns regarding methodological limitations for the body of data contributing to each review finding. In practice, minor concerns will not lower our confidence in the review finding, while serious concerns will lower our confidence. Moderate concerns may lead us to consider lowering our confidence in our final assessment of all four CERQual components.

Where you have concerns about methodological limitations, describe these concerns in the CERQual Evidence Profile in sufficient detail to allow users of the review findings to understand the reasons for the assessments made. The Evidence Profile presents each review finding along with the assessments for each CERQual component, the overall CERQual assessment for that finding and an explanation of this overall assessment. For more information, see the second paper in this series [7].

### Examples of assessing methodological limitations

In Table 1, we give examples of how methodological limitations can be assessed for a selection of review findings. These examples illustrate how different, and differing degrees of, methodological weaknesses can affect the overall assessment of methodological limitations for a review finding.

### Implications when methodological limitations are identified

Concerns about methodological limitations may not only have implications for our confidence in a review finding but can also point to ways of improving future research. Firstly, where serious methodological limitations have been identified, this may indicate the need for future primary researchers to use more appropriate methods or to report their methods more clearly. You should also consider updating the review once this research is available.

## Conclusions

Concerns regarding methodological limitations may lower our confidence in review findings and are therefore part of the CERQual assessment. However, it is also important to remember that this is just one component of the CERQual approach. Having concerns about methodological limitations may not necessarily lead to a downgrading of overall confidence in a review finding, as this will be assessed alongside the other three CERQual components.

In this paper, we have described how the methodological limitations component have been used so far and have provided guidance to review authors and others on how to assess this component. However, more work is needed to determine which criteria critical appraisal tools should include and to explore how different methodological weaknesses might impact upon an overall assessment of confidence. We currently recommend that whichever tool is used, you provide a transparent description of their assessments of methodological limitations and how this impacts our confidence in a review finding. We expect the methodological limitations component, as well as the CERQual approach more generally, to develop as we gain experience and feedback from increased practical application of the approach.

## Open peer review

Peer review reports for this article are available in Additional file 2.

## Additional files

Additional file 1: Key definitions relevant to CERQual. (PDF 619 kb)

Additional file 2: Open peer review reports. (PDF 99 kb)
